# Native Chemical Ligation of Peptoid Oligomers

**DOI:** 10.1021/acs.biochem.5c00833

**Published:** 2026-03-16

**Authors:** Matthew R. Seraydarian, Michael D. Connolly, Ronald N. Zuckermann, Kent Kirshenbaum

**Affiliations:** 1 Department of Chemistry, 5894 New York University, 100 Washington Square East, New York, New York 10003, United States; 2 The Molecular Foundry, 1666 Lawrence Berkeley National Laboratory, 1 Cyclotron Road, Berkeley, California 94720, United States

## Abstract

Bioorganic chemists are inspired by natural biopolymers to design peptidomimetic oligomers that can exhibit sequence-structure–function relationships. Biomimetic polymers can be synthesized to incorporate a specific sequence of nonbiological monomer units using a variety of iterative solution-phase or solid-phase reaction schemes. These protocols generally provide access to a vast diversity of oligomeric compounds but are limited with respect to their ability to attain protein-like chain lengths. This constraint can preclude access to sequence-defined synthetic macromolecules with sufficient sizes required to exhibit tertiary structure and other protein-mimetic attributes. In contrast, peptide chemists have overcome this limitation by developing convergent synthetic methods, such as native chemical ligation, to join individual, smaller peptide chains together to make larger peptides or full proteins. A similar convergent approach is needed to establish efficient synthetic routes to non-natural sequence-defined macromolecules. Herein, we adapt the peptide native chemical ligation method to peptoid oligomers, demonstrating how short chains can be conjoined to create sequence-defined peptoid macromolecules. Nanosheet-forming peptoid polymers with distinct surface loop display domains were generated by sequential ligation of several discrete fragments. This method provides a reliable convergent ligation route for sequence-defined polypeptoids that results in a native amide bond joining the fragments. We envision that this strategy will be useful in synthesizing peptoid-based proteomimetics that incorporate diverse chemical features.

## Graphical abstract

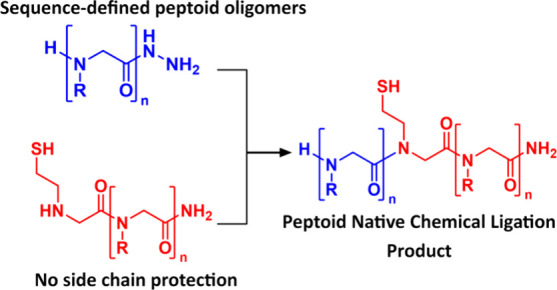

## Introduction

Nature’s inspiration in the field of synthetic chemistry and polymer science is particularly evident in the pursuit of sequence-defined macromolecules.^1–3^ The sequence-structure–function paradigm of natural biopolymers such as proteins inspires researchers to attempt to recapitulate this relationship in synthetic systems.^1–3^ Functional biopolymers typically feature strict control of the sequence of their monomer units. The sequence specificity is considered a requirement to encode self-directed folding into hierarchical structures.^4–7^ In addition, macromolecular biopolymers exhibit substantial chain lengths sufficient to attain tertiary structures that enable attributes such as enzymatic catalysis, allosteric regulation, selective molecular recognition, and other sophisticated functions. Biomimetic chemists have made strides toward creating synthetic oligomeric mimics of biopolymers using abiotic monomer types, which in many cases are capable of folding into well-ordered secondary structures.^8–15^ Substantial progress has been made in the synthesis of these sequence-defined “foldamer” molecules.^16^ Despite the growth in the field of sequence-defined macromolecules, much less progress has been made in attaining both sequence-specificity and macromolecular chain lengths in synthetic systems.

Sequence-defined foldamers include a broad range of peptide structural analogs, including beta peptides, gamma peptides, oligoureas, and peptoids.^8–15^ Peptoids are protein-inspired, N-substituted glycine oligomers and have drawn the attention of the foldamer, materials, nanoscience, and polymer physics communities due to their ease of synthesis, chemical stability, and capability of forming a range of secondary structures and nanostructures. Sequence-defined peptoids are synthesized by an iterative solid phase submonomer method (Scheme 1).^17,18^ Each monomer addition reaction involves two steps: bromoacetylation of a resin-bound amine, followed by displacement of the bromine with a primary amine.^17,18^ The use of primary amine synthons offers convenient and economical access to an extraordinary chemical diversity of peptoid side chains.

**Scheme 1:**
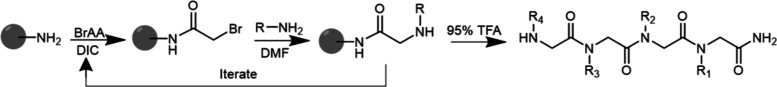
General Peptoid Submonomer Synthesis Protocol

Peptoids prepared via solid phase synthesis are subject to diminishing yields as chain length increases. Automated solid phase protocols have made the synthesis of sequence-specific peptoids at lengths of around 50 monomers feasible. However, reaching chain lengths beyond ∼50mers remains challenging, particularly for sequences that may include relatively low-yielding monomer types. As chain length increases, the presence of deletion products increases as well, which complicates the purification of long chains. Peptoid chains may also be synthesized via solution phase synthetic techniques. Recent work from the Tran lab demonstrated the synthesis of partially sequence-controlled peptoid of up to 32 monomer units via iterative exponential growth (IEG).^19^ In addition, peptoid homopolymers or random copolymers can be generated via ring opening polymerization of N-substituted N-carboxyanhydrides, but these products retain little sequence specificity.^20,21^ Thus, the current choice of peptoid synthesis protocols offers a stark dichotomy between accessing macromolecular chain lengths or attaining sequence specificity.^22^

In the case of polypeptide chemical synthesis, the use of fragment ligation techniques has largely overcome this trade-off. The common strategy in this arena is native chemical ligation (NCL).^23,24^ NCL relies on the selective reaction between a peptide fragment bearing an N-terminal cysteine and another peptide bearing a synthetically prepared C-terminal thioester (Scheme 2). The reaction results in a native amide bond with a cysteine residue at the ligation site and proceeds without requiring side chain protecting groups on either peptide fragment.^23,24^ Peptides used in NCL reactions are frequently prepared by convenient solid phase synthesis. Thus, the synthesis and ligation of two or more fragments can overcome the limitation of diminishing yields at substantial chain lengths.^24^ Because NCL results in a native peptide bond at the ligation site, no additional chemical moieties are introduced via ligation that could alter folding or biocompatibility. NCL has enabled the complete chemical synthesis of proteins and offers an ability to install noncanonical amino acids within protein chains.^23,25^

**Scheme 2:**
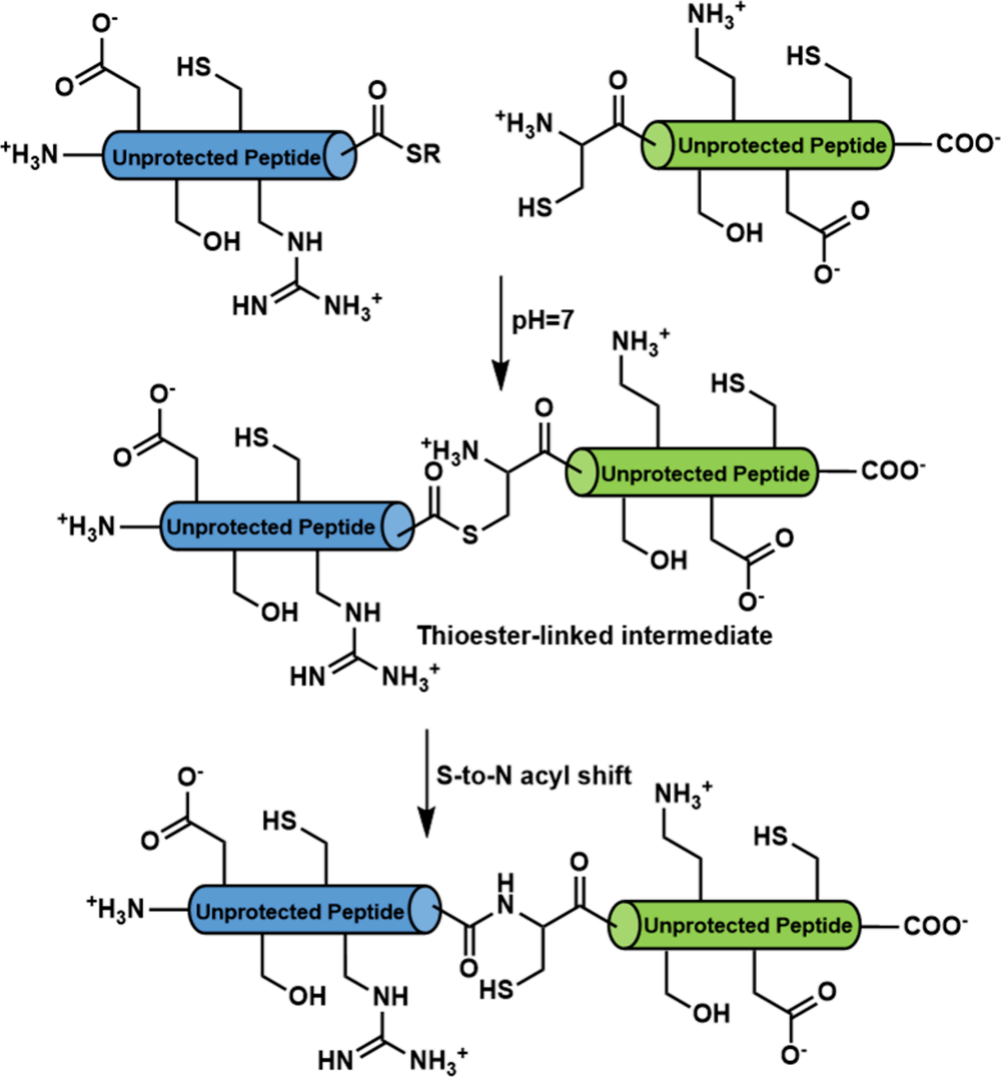
Canonical Peptide Native Chemical Ligation

There are only limited examples of proteomimetic sequence-defined macromolecular systems utilizing any type of ligation chemistry as a method to achieve extended chain lengths. Nevertheless, the ambitious development of functional protein mimics will demand new strategies such as fragment condensation or programmed self-assembly in order to access well-defined tertiary and quaternary structures. A critical requirement for ligation is to identify reaction conditions that are orthogonal to many reactive functional groups present in the oligomer fragments. Copper catalyzed azide–alkyne cycloaddition (CuAAC) has been used to link “aryl-triazole-amide” units into larger foldamer sequences.^26^ In the case of oligourea compounds, helical oligomers were joined covalently via turn motifs.^27,28^ Oligoureas could also be self-assembled with oligoamides via hydrogen bonding networks.^27,28^ Although there have been a handful of other examples of ligation strategies, none has been as versatile or efficient as NCL implemented on peptides.^29–32^

Peptoid chemists have long sought a reliable oligomer ligation method like NCL. Previous efforts to form covalent linkages between peptoid fragments have involved various conjugation strategies. A prominent example of a structured peptoid macromolecule used orthogonal oxime and disulfide linkages to connect peptoid helices.^8^ Although this work yielded a biomimetic multihelical bundle, the backbone introduced non-native backbone linkages between monomers. To study the impact of hydrophobic-polar patterning on the folding of proteomimetic polymers, peptoid 100mers were synthesized by linking peptoid 50mers together via CuAAC.^33^ While an accomplishment in the preparation of a sequence-defined peptoid macromolecule, a non-native linkage was again used to join the fragments.^33^ Previous work from our research group demonstrated the protease catalyzed ligation of peptoid oligomers.^34^ Although peptoid sequences with molecular weights of up to 20 kDa were obtained, the products were polydisperse and the sequences contained only unreactive side chains.^34^ We also demonstrated the use of serine/threonine ligation (STL) to synthesize peptoid/peptide and peptoid/protein hybrids.^35,36^ For STL, a peptoid bearing a C-terminal salicylaldehyde ester was prepared and ligated to a peptide or protein bearing an N-terminal serine residue. However, the preparation of the salicylaldehyde ester required that the peptoid fragment bear no reactive side chain types, significantly limiting its monomer composition.^35,36^ A peptoid ligation method that preserves the backbone and is compatible with unprotected peptoid oligomers has heretofore eluded the peptoid community. A long-standing obstacle to achieving this objective is that peptoid C-terminal thioesters cannot be prepared by traditional submonomer solid phase synthesis protocols. C-terminal thioesters are not stable in basic conditions, thus the use of primary amines as synthons in peptoid submonomer synthesis has hampered the creation of peptoid C-terminal thioesters. NCL has therefore proven unsuitable for constructing sequence-defined peptoid macromolecules. Other popular chemical ligation techniques such as STL and KAHA ligation are not readily compatible with ligating sequences consisting entirely of peptoid fragments.^37–40^

A recent development in peptide NCL is the use of C-terminal hydrazides as masked thioesters.^41–44^ Developed by the Liu lab, the hydrazide ligation technique involves converting the C-terminal hydrazide to a thioester *in situ* just before initiating the ligation.^41–43^ Preparing a C-terminal hydrazide rather than a thioester allows for the peptide chain to be prepared in basic conditions.^41–43^ We hypothesized that this ligation technique may allow for C-terminal functionalized peptoid chains to be synthesized for subsequent NCL reactions. In this work, we report the use of the hydrazide ligation technique for native chemical ligation of peptoid oligomers (Scheme 3). We successfully ligated multiple small peptoid oligomers together; we used this ligation chemistry as a method for peptoid macrocyclization; and we demonstrated the modular synthesis of peptoid sequences up to 38mers that are capable of self-assembly into 2-dimensional nanosheets.

**Scheme 3:**
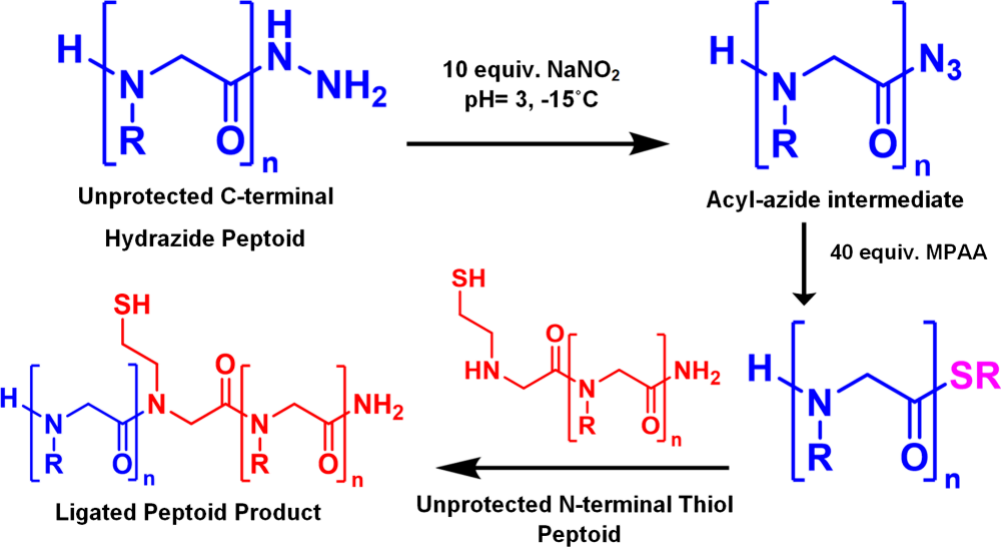
C-Terminal Peptoid Hydrazide Oxidation, Thiolysis, and Ligation

## Materials and Methods

Bulk solvents (N,N-dimethylformamide, dichloromethane, acetonitrile, etc.) were purchased from VWR. All reagents for synthesis including primary amines, amino acids, coupling agents, and ligation materials were purchased from Millipore Sigma, Chem-Impex, Thermo Fisher Scientific, or Ambeed.

Peptoid oligomers were synthesized either manually or on an automated synthesizer implementing the solid phase submonomer method.^17^ Peptoid C-terminal hydrazides were prepared from a hydrazine resin that is obtained from 2-chlorotrityl chloride resin according to the procedure developed by Bird and Dawson.^45^ The C-terminal residue of the peptoid hydrazide was incorporated using standard Fmoc amino acid coupling (5 eq amino acid, 4.9 eq HCTU, 10 eq DIPEA), followed by peptoid submonomer chemistry (10 eq bromoacetic acid, 12 eq DIC for coupling, 15 eq primary amine for displacement) for the rest of the oligomer. Peptoids containing an N-terminal thiol were cleaved in 92.5% trifluoroacetic acid, 2.5% water, 2.5% triisopropylsilane (TIPS), and 2.5% 1,2 ethanedithiol. All other peptoids were cleaved from solid support in 95% TFA, 2.5% water, and 2.5% TIPS. Crude peptoid products were analyzed via LCMS and analytical HPLC or UPLC-MS, and peptoids were purified via preparative reverse-phase HPLC.

Peptoids composed of six or fewer monomers were purified on a Jupiter C18 21.2 × 250 mm column. Nanosheet-forming peptoid and loop forming sequences were purified on a Waters XBridge BEH300 Prep C18 19 × 100 mm column. Peptoids were purified using acetonitrile and water as mobile phases, with each phase containing 0.1% TFA. The identities of crude and pure peptoids were confirmed via mass spectrometry, either on an Agilent 6120 LCMS-SQ or Waters Acquity UPLC with Waters SQD2 single quadrupole mass spectrometer.

### Synthesis of 3-(Tritylthio)-propylamine

Tritylmethanethiol (1.382 g, 5.0 mmol, 1 equiv) was dissolved in 10 mL DMF in a round-bottom flask. The flask was cooled to 0 °C and 1.203 g (11.0 mmol, 2.2 equiv) triethylamine was added dropwise. The mixture was kept stirring at 0 °C for 10 min. 3-bromopropylamine hydrobromide (1.096 g, 5.0 mmol, 1 equiv) was dissolved in 5 mL DMF and the solution was added dropwise over 5 min into the flask at 0 °C. The mixture was warmed to room temperature and stirred overnight. The reaction was monitored by TLC (DCM: MeOH = 5:1). The reaction was extracted with water and ethyl acetate. The organic layer was washed with water twice and dried over sodium sulfate. The dried solution was filtered and concentrated via rotary evaporation. The residue was purified by column chromatography (eluting with DCM: MeOH = 20:1). The product was obtained as a brown solid (700 mg, 2.10 mmol, 42% yield),

^1^H NMR (400 MHz, CDCl_3_): δ 1.59 (p, 2H, CH2-CH2-CH2, J = 8.0 Hz), 2.24 (t, 2H, CH2-S, J = 8.0 Hz), 2.42 (s, 2H, NH2), 2.68 (t, 2H, CH2-NH2, J = 8.0 Hz), 7.21–7.26 (m, 3H, aryl-H), 7.28–7.34 (m, 6H, aryl-H), 7.43–7.47 (m, 6H, aryl-H) ppm (SI Figure 1).

### General Strategy for Native Chemical Ligation of Peptoids

A general strategy for native chemical ligation of peptoids was modified from analogous protocols for ligating peptide hydrazides.^42^ C-terminal hydrazide peptoid (2 μmol) was dissolved in 0.4 mL of ligation buffer (0.2 M NaH_2_PO_4_, 6 M Gn·HCl, pH = 3) in a 1.7 mL microfuge tube. The peptoid bearing the N-terminal thiol (2 μmol, 1 equiv) and 40 eq of 4-mercaptophenylacetic acid (MPAA) were dissolved in 0.4 mL of ligation buffer in a separate 1.7 mL microfuge tube and the pH was adjusted to 6.5. Once all solid materials were completely dissolved, the tubes containing the peptoid solutions were placed in a −15 °C ice/salt bath. To the solution containing the C-terminal peptoid hydrazide, 10x excess of 0.5 M aqueous NaNO_2_ (40 μL of 0.5 M NaNO_2_ solution for 2 μmol scale) was added and the tube was slightly agitated in the ice/salt bath for 15 min. The two peptoid solutions were then mixed, warmed to room temperature, and the resulting solution was neutralized to pH 6.8–7. The solution was placed on a shaker overnight and analyzed via analytical HPLC and LCMS or UPLC-MS. The percent conversion of the starting material to the ligated product was determined by analytical HPLC or UPLC and calculated from the disappearance of the starting material peaks and appearance of a product peak. If necessary, ligated peptoids were purified via preparative or semipreparative reverse-phase HPLC. Prior to analysis, purification, or nanosheet formation, ligated peptoid solution was treated with 0.2 M TCEP dissolved in ligation buffer to reduce any disulfide products.

### Radical Desulfurization

Ligation product peptoid H-Npm-(Npl)_3_-Sar-Nte-(Npl)_3_-NH_2_ (4.7 mg) was dissolved in 0.2 mL neutral ligation buffer (same buffer as used in ligation, but pH adjusted to 6.9–7.0) in a 1.7 mL microfuge tube. To the same microfuge tube, 0.2 mL of 1 M TCEP dissolved in neutral ligation buffer, 100 μL of tBuSH, and 50 μL of 0.1 M 2,2’-azobis­[2-(2-imidazolin-2-yl)­propane] dihydrochloride (radical initiator) dissolved in neutral ligation buffer were added. This combination was placed on an incubated shaker at 37 °C for four hours. After four hours, the reaction was analyzed via analytical HPLC and LCMS.

### Peptoid Macrocyclization Using NCL Chemistry

Linear peptoid precursor (1.4 mg) was dissolved in 1 mL of ligation buffer (0.2 M NaH_2_PO_4_, 6 M Gn·HCl, pH = 3). A 10x excess of 0.5 M aqueous NaNO_2_ was added to the dissolved peptoid solution at −15 °C for 15 min. MPAA (40x excess) was dissolved in 0.4 mL of ligation buffer and added to the peptoid solution (for a final peptoid concentration of 1 mg/mL) and the pH was adjusted to 6.8–7. The reaction progressed overnight and was analyzed via analytical HPLC and LCMS the following day.

### Peptoid Nanosheet Formation

A stock solution of nanosheet-forming buffer was prepared (10 mM Tris-HCl, 100 mM NaCl, pH 8 in water). A sample of ligated peptoid in ligation buffer was diluted into nanosheet formation buffer such that the final concentration of peptoid in nanosheet formation buffer was 20 μM. The vial of peptoid in nanosheet formation buffer was held in a horizontal position for 15 min, rotated into a vertical position for a few seconds, and rotated back into the horizontal position. This rocking process was repeated for 3 days.

### Fluorescence Imaging of Assembled Peptoid Nanosheets

Nanosheets were imaged using fluorescence microscopy using the environmentally sensitive dye, Nile Red. Nile Red (Thermo Fisher Scientific) was solubilized in DMSO as a stock solution at 1 mM. Stock solutions were then diluted to 0.1 mM in Milli-Q water and then used a final concentration of 1 μM in a 10 μL solution containing assembled nanosheets.

For imaging, agarose (Sigma-Aldrich) was added to Milli-Q water at final concentration of 2% (w/v). After heating until the solution was molten, 700 μL was dropped onto a clean glass slide (Globe Scientific) and then flattened by adding another glass slide on top. After solidifying, the slides were separated, and 1 μL of stained nanosheets were added to the agarose pad.

Nanosheets on the agarose pads shown in Figure 2 were imaged on a Zeiss LSM710 confocal microscope using a 10*x*/0.3 NA Plan-Neofluar objective. Nile Red was excited using a 561 nm DPSS laser at 16% of the total power (measured at 0.47 mW out of the objective). The excitation beam was separated from the emitted light using a 488/561 nm beam splitter, with the emitted light then passed through a pinhole set to 71 μm and imaged on a PMT detector at 0.79 μs with the prism set to a wavelength of range set to 563–753 nm to capture the main emission peak of Nile Red.

The nanosheets shown in Figure 3 were imaged on an inverted Zeiss Elyra 7 microscope using a Plan-Apochromat 20*x*/0.8 NA objective (Zeiss) and an MBS 405/488/561/641 and EF LBF 405/488/561/641 filter set, and an LP 560 and a BP 570–620 + LP 655 filter cubes. Nile Red was illuminated using a 0.5 W Sapphire 561 nm laser (Coherent) at 10% of the total power (measured at 100 mW out of the objective) in epi-fluorescent mode. Data was split on a Duolink system using a 570–620 + LP655 filter and imaged on a pco.edge 4.2 high speed sCMOS camera (1000 ms exposure). Images from both microscopes were then processed and false-colored using Zeiss Zen Black.

## Results and Discussion

The development of NCL for peptoids required the generation of oligomers incorporating C-terminal hydrazides, along with partner oligomers bearing a side chain thiol at the N-terminus. We initially attempted to generate small peptoid oligomers with C-terminal hydrazides from the prepared hydrazine resin using peptoid solid phase submonomer synthesis throughout. No peptoid products were detected when using this strategy, suggesting that the bromoacetic acid/DIC coupling conditions were incompatible for initiating oligomer synthesis from the hydrazine resin. We then explored initiating synthesis with an *N*-methylglycine (sarcosine) unit as the C-terminal peptoid residue, allowing us to use protected N-Fmoc sarcosine as our first synthon and employing standard peptide coupling conditions for the first monomer addition. Standard peptoid submonomer coupling cycles could then be used for each ensuing residue, successfully yielding the desired peptoids. For solid phase peptide synthesis, it is not uncommon to employ alternative coupling conditions for the addition of the first amino acid to similar resin linkers.^46^

We first demonstrated the ligation reaction on short model sequences: H-Npm-(Npl)_3_-Sar-NHNH_2_ (peptoid **1**) and H-Nte-(Npl)_3_-NH_2_ (peptoid **2**). Cysteamine was chosen as the thiol-bearing primary amine to retain the critical five-membered ring intermediate characteristic for canonical peptide NCL.^23,24^ We postulated that the mechanism of NCL between two peptoids could then follow a mechanism nearly identical to that of traditional peptide NCL, with the only difference being the S→N acyl shift involving a nucleophilic attack performed by a secondary amine rather than a primary amine. We adapted the protocol of peptide hydrazide ligation developed by the Liu lab for the ligation of our peptoids, conducting the hydrazide oxidation in aqueous buffer at pH 3, and the subsequent ligation in aqueous buffer at pH 7 (Scheme 3). Upon analytical HPLC and LCMS analysis, a new major product peak was observed with a mass corresponding to the desired ligation product, peptoid **3** (calc. *m*/*z*[M + H]: 947.5 observed *m*/*z*[M + H]: 947.2 calc. *m*/*z*[M + Na]: 969.5 observed *m*/*z*[M + Na]: 969.2) (Figure 1).

**Figure 1:**
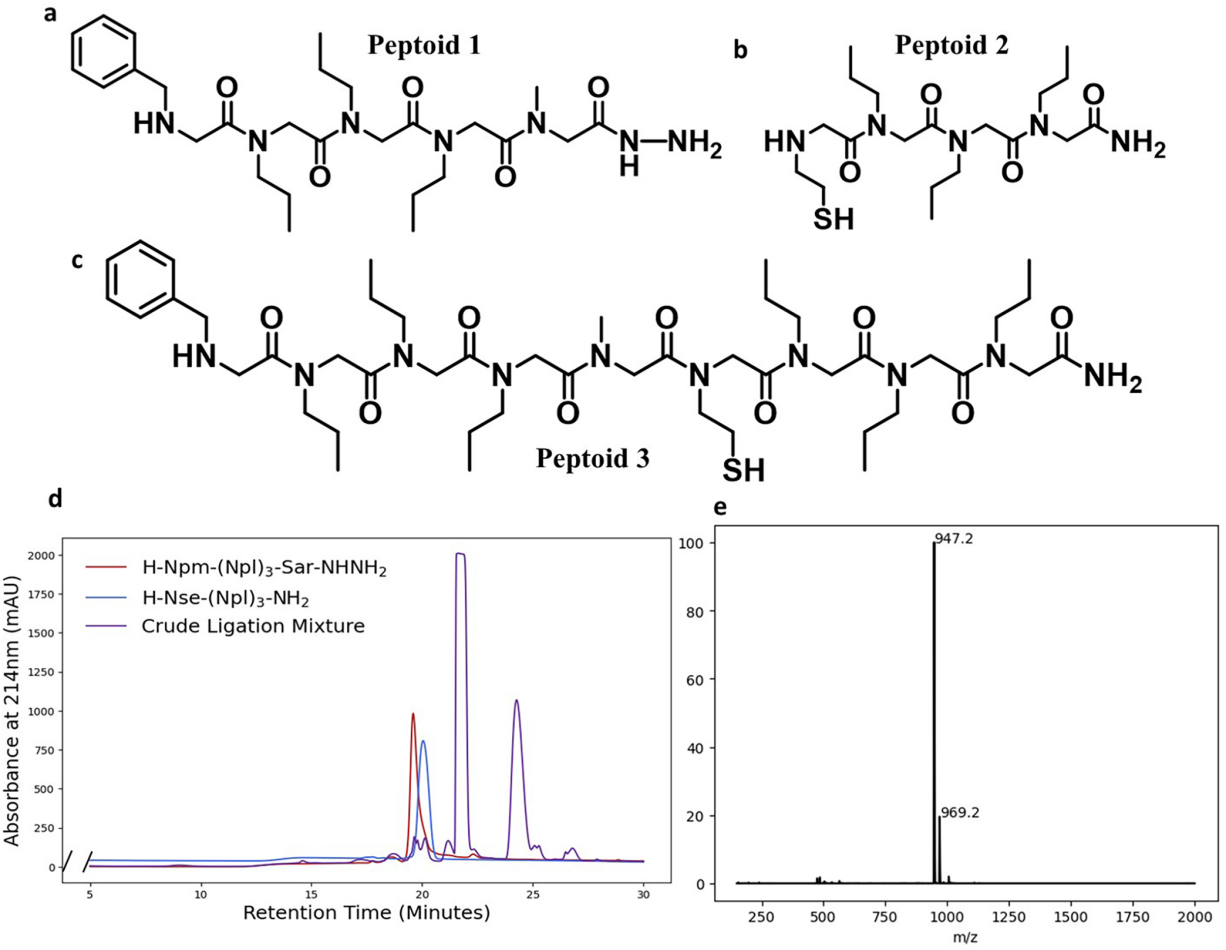
(a) Peptoid **1** H-Npm-(Npl)_3_-Sar-NHNH_2_, the C-terminal hydrazide peptoid fragment used in first ligation (b) Peptoid **2** H-Nte-(Npl)_3_-NH_2_, the N-terminal thiol-bearing peptoid fragment used in first ligation (c) Sequence of peptoid **3** following a successful NCL reaction of peptoids **1** and **2**. (d) Analytical HPLC traces of the crude ligation mixture overlaid with the traces of the pure starting material peptoids. The peak at ∼24 min contained the product. The large peak at ∼22 min contains the thiol additive MPAA, which is used in excess. (e) Positive ion mode mass spectrum of the selected product peak displaying the expected [M + H] and [M + Na] peaks.

Following this successful initial ligation reaction, we tested the scope of the reaction by varying some of the side chain chemical functional groups present in the oligomers used in ligation (Tables 1 and 2). For convenience, sarcosine was most frequently incorporated as the C-terminal monomer for hydrazide peptoids used in ligations. We also demonstrated that N-benzylglycine, another peptoid monomer commercially available in its N-Fmoc form, can also be used as the C-terminal monomer on the hydrazide peptoid (peptoid **4**) and participate in ligation with peptoid **5** to form the expected product (peptoid **6**).

**Table 1:**
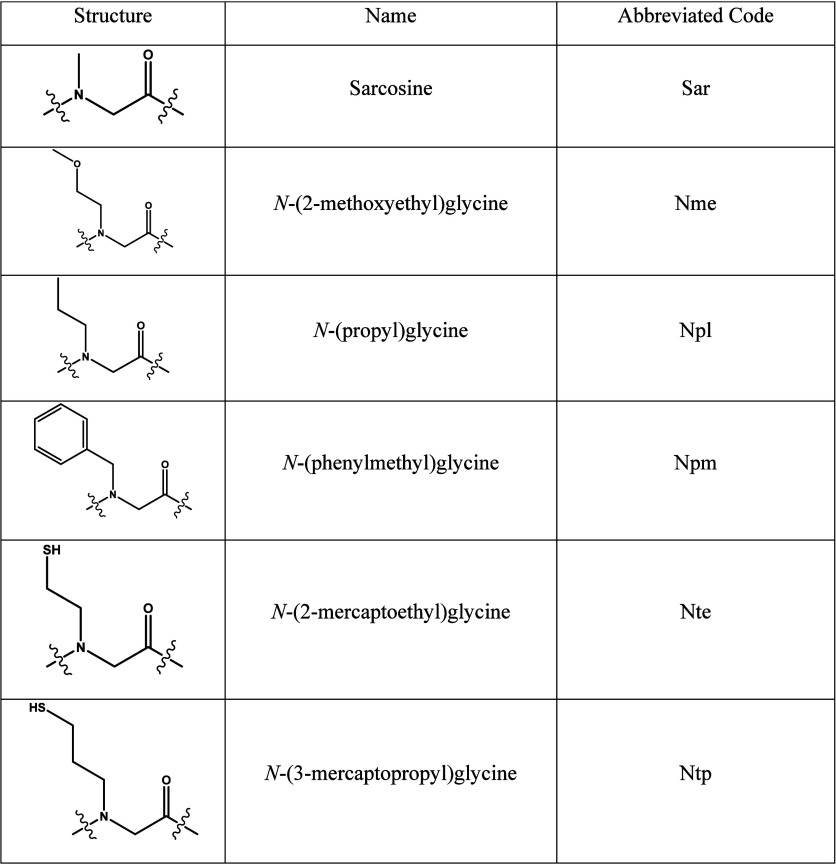
Structures and Abbreviations of Peptoid Monomer Types Used in This Study

**Table 2:** Model Oligomers Ligated and Corresponding Products

C-terminal hydrazide sequence **(**peptoid **#)**	N-terminal thiol sequence **(**peptoid **#)**	Ligated product sequence **(**peptoid **#)** calculated mass: observed mass
H-(Npl-Npm)_2_-NHNH_2_ **(4)**	H-Nte-(Npl)_3_-NH_2_ **(5)**	H-(Npl-Npm)_2_-Nte-(Npl)_3_-NH_2_ **(6)** 924.2: 924.8
H-Npm-(Nme)_3_-Sar-NHNH_2_ **(7)**	H-Ntp-Npl-Npm-Nme-NH_2_ **(8)**	H-Npm-(Nme)_3_-Sar-Ntp-Npl-Npm-Nme-NH_2_ **(9)** 1025.3: 1024.8
H-Npm-(Nme)_3_-Sar-NHNH_2_ **(7)**	H-Nte-Npl-Nte-Npm-Nme-Npl-NH_2_ **(10)**	H-Npm-(Nme)_3_-Sar-Nte-Npl-Nte-Npm-Nme-Npl-NH_2_ **(11)** 1227.6: 1226.8

To understand how variations in the N-terminal thiol containing monomer could be tolerated, we synthesized peptoid **7**, containing an N-terminal S-Trt-3-mercaptopropylglycine, which is one methylene group longer than the commercially available Trt-cysteamine we most frequently used. Previous work has demonstrated using synthetic cysteine analogs as the reactive thiol species in peptide NCL, including analogs that lengthen the distance between the backbone and thiol.^47–50^ When using peptoid **7** in a ligation reaction with peptoid **8** containing the 3-mercaptopropyl side chain at the N-terminus, we observed the expected ligation product (peptoid **9**). We used peptoid **7** with a peptoid containing an internal thiol in addition to the N-terminal thiol (peptoid **10**) in ligation and observed that ligation proceeded in the presence of the internal thiol (peptoid **11**). The product formation of each of these ligation reactions was confirmed via LCMS (SI Figure 2). In general, we observed good conversion to ligated products for each of these initial oligomer ligations, with some conversions approaching 90%. In order to establish a representative isolated yield for these initial oligomer ligations, we purified the product of the ligation between peptoids **1** and **2** to obtain the product peptoid **3**. A 32% yield was obtained following purification via semipreparative HPLC. After isolating the purified ligation product, we subjected peptoid **3** to radical desulfurization to remove the thiol functionality. Radical desulfurization is a common strategy when using NCL for the total chemical synthesis of proteins to convert the required cysteine residue to an alanine, which is more stable and naturally abundant.^51,52^ Analytical HPLC and LCMS revealed the detection of the desulfurized product (peptoid **3DS**), now incorporating an N-ethylglycine at the ligation site. (calc. *m*/*z*[M + H]: 914.6 observed *m*/*z*[M + H]: 915.0) (SI Figure 3).

We then used NCL protocol as a method for macrocyclization of a peptoid sequence bearing both an N-terminal thiol and a C-terminal hydrazide on a single peptoid hexamer. Following oxidation of the C-terminal hydrazide of the linear peptoid **12** bearing an N-terminal thiol, the cyclization was initiated via the addition of MPAA (Scheme 4). Analytical HPLC and LCMS revealed near-complete conversion of the linear precursor to the cyclic product peptoid **13** after an overnight reaction (SI Figure 4).

**Scheme 4:**
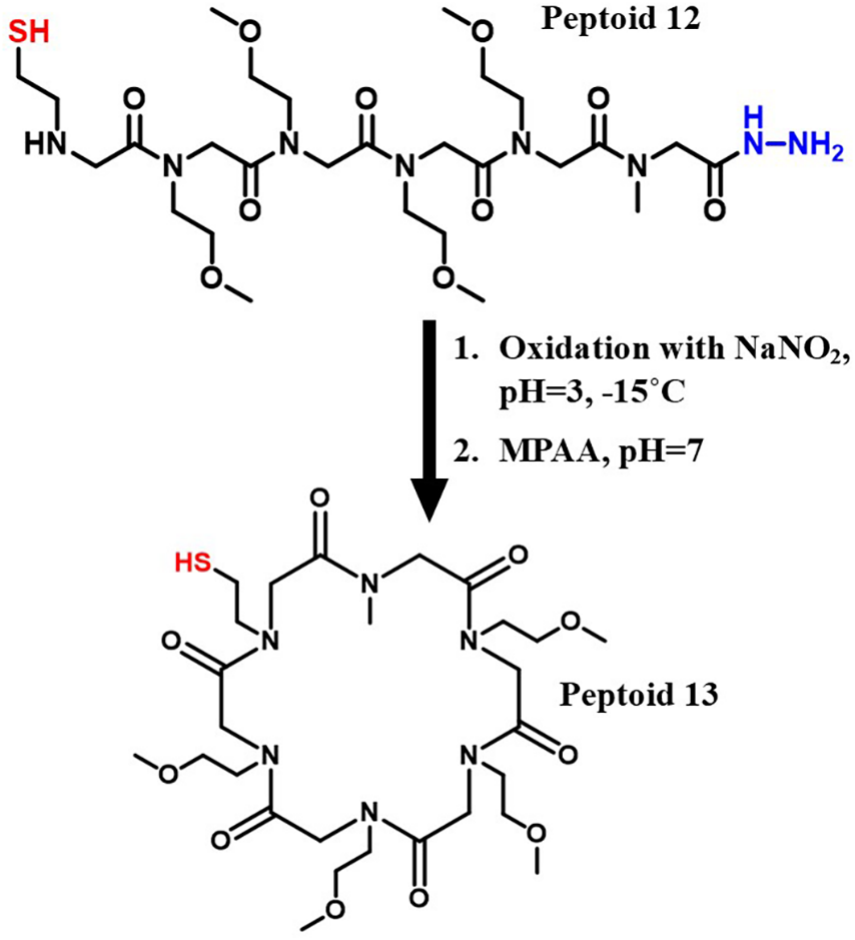
NCL Chemistry as a Method for Peptoid Macrocyclization

To demonstrate further applications of this reaction, we used this ligation as a method to prepare peptoid-based nanomaterials. Peptoid nanosheets are 2D nanomaterials comprising nonbiological polymers that have been extensively developed to enable a range of functional attributes.^53^ For example, previous work has established that large peptoid nanosheets can be synthesized with loops projected from their hydrophilic surfaces to effectuate molecular recognition.^53–56^ Individual peptoid chains self-assemble into robust peptoid nanosheets via alignment of the oligomers within a monolayer at the air–water interface. The subsequent collapsing of these chains via gentle agitation and compression of the air–water interface yields the 2D nanosheets.^53,57^ Peptoid nanosheets displaying functional loops frequently require synthesis of constituent peptoid oligomers with substantial chain lengths.^54–56^ The synthesis of loop-bearing peptoid nanosheets may therefore benefit greatly from a fragment condensation method like NCL. Not only would NCL allow for the preparation of shorter peptoid oligomers resulting in increased yields, but a modular ligation strategy provides a streamlined method for connecting different sequences together. Thus, we designed peptoid sequences that could be ligated to form a loop-presenting peptoid nanosheet-forming sequence. The design of the peptoid oligomer fragments to be ligated was such that the thiol functional group that is necessary for ligation would be present in the loop after ligation. We synthesized multiple variations of peptoid nanosheet-forming sequences via NCL. We initially synthesized peptoids **14** and **15**, with multiple positively charged side chain groups within the N-terminal block and the negatively charged side chain groups within the C-terminal block, following conventional peptoid nanosheet design strategies (Figure 2). A ligation product was identified via analytical HPLC and mass spectrometry. A sample of the ligation solution was diluted into nanosheet formation buffer and rocked to form the desired peptoid nanosheets. The nanosheet products were visualized via fluorescence microscopy (Figure 2).

**Figure 2:**
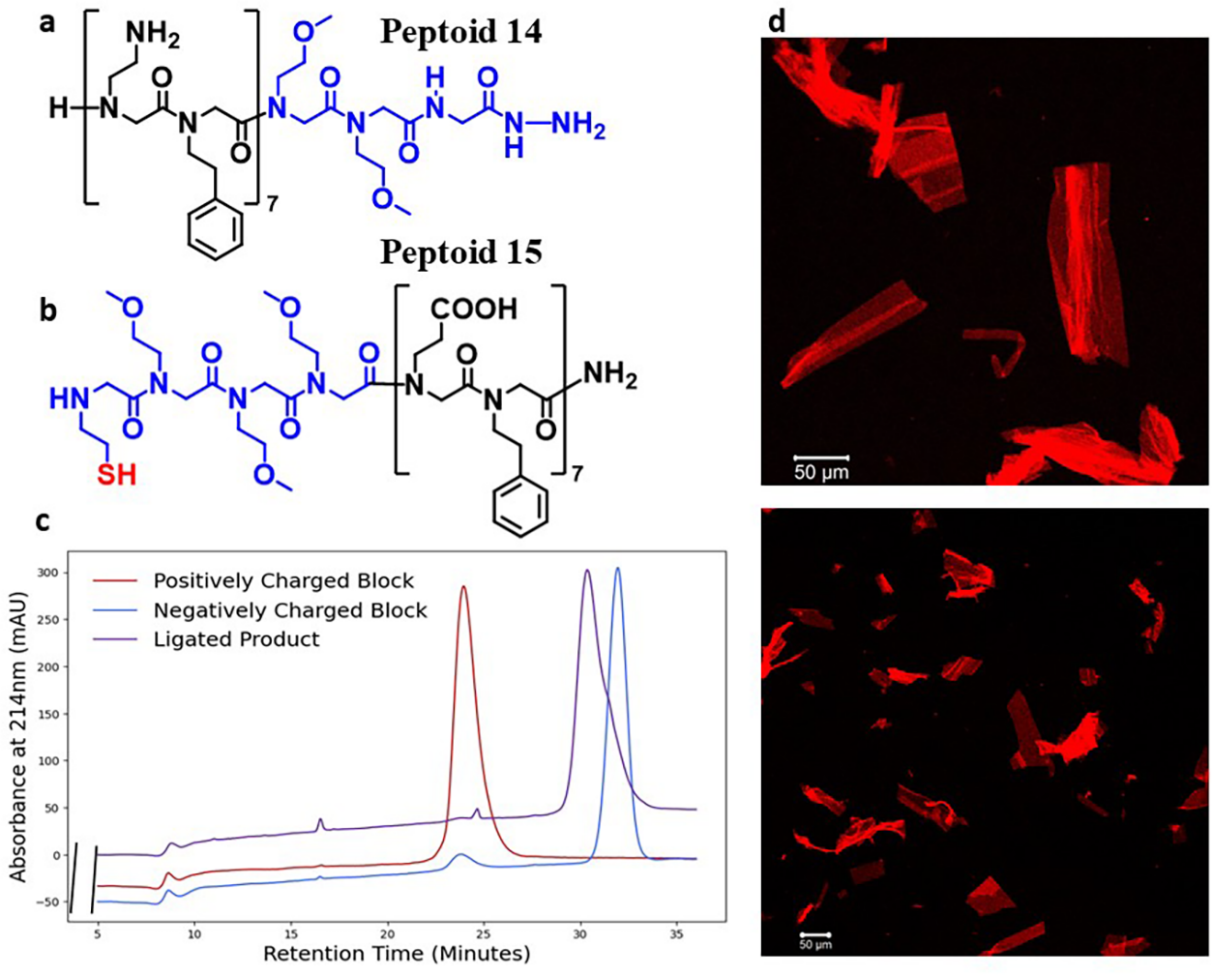
Evidence of the formation a nanosheet-forming product from the ligation of peptoids **14** and **15** (a) and (b) Sequences of peptoids **14** and **15,** respectively. The loop segment is depicted in blue. (c) Analytical HPLC traces of pure peptoids **14** and **15**, and the purified ligation product of the two (d) Fluorescence microcopy imaging of micron-scale peptoid nanosheets formed from the ligation product of peptoids **14** and **15**. Nanosheets were stained with Nile Red dye and imaged on a Zeiss LSM710 confocal microscope.

Unexpectedly, the molecular weight of the major ligation product obtained was 29 Da larger than calculated (SI Figure 5). Upon further analysis it was determined that this adduct was formed during the oxidation of the N-terminal positively charged fragment, and not during the subsequent ligation step. It is known that secondary amines may react with the oxidative NO^+^ species generated from NaNO_2_ in acidic conditions to form an N-nitrosamine.^58^ We hypothesize that during the oxidation of a peptoid bearing an amine-functionalized N-terminal side chain with aqueous NaNO_2_ in acidic conditions, the terminal secondary amine is converted to an N-nitrosamine. An N-nitrosamine at the N-terminus of the expected ligation product would account for the additional mass. This phenomenon was not seen in any previous ligations we conducted, substantiating our hypothesis that the N-nitrosamine is stabilized by the presence of the primary amine within the N-terminal side chain and formation is enhanced by the presence of a neighboring aminoalkyl side chain. Dipeptides with an N-terminal proline have been reported to form an N-terminal N-nitrosamine when treated with NaNO_2_ in acidic conditions.^59^ To further elucidate this phenomenon, we conducted a detailed study of the N-nitrosamine formation in model peptoid systems (see SI including SI Figures 6–8).

To further confirm our hypotheses and determine whether other nanosheet-forming sequences may be subject to the same phenomenon, we synthesized peptoids **16** and **17** (see Figure 3). The ligation product of these sequences is a nanosheet-forming peptoid with the opposite charge orientation as previously synthesized (i.e., the negatively charged side chains are within the N-terminal block and the positively charged side chains are within the C-terminal block). These sequences were ligated and the resulting product peptoid **18** was gently agitated, which facilitated its assembly into nanosheets (Figure 3). The identity of peptoid **18** was determined via UPLC-MS (SI Figure 9). The molecular weight of this ligation product was as expected, with no evidence of +29 Da contaminating species. The nanosheets formed from these ligated sequences were similar to loop-presenting peptoid nanosheets synthesized in previous studies.^54–56^ The estimated percent conversion to ligated products for the ligations of nanosheet-forming sequences was generally good as well, in the range of 50–70% by analytical UPLC. We conducted a kinetic study of the ligation of peptoids **16** and **17** to peptoid **18** and found that the majority of the product is formed within 12 h. During this study, we observed that the thioesterified intermediate formed from peptoid **16** is hydrolyzed over the course of the reaction, quenching its ability to participate in ligation. It is clear that the efficiency of peptoid NCL is dependent on multiple factors, including peptoid sequence and concentration. Such factors likely contributed to variations in conversions observed in this study and suggest an opportunity for future optimization.

**Figure 3:**
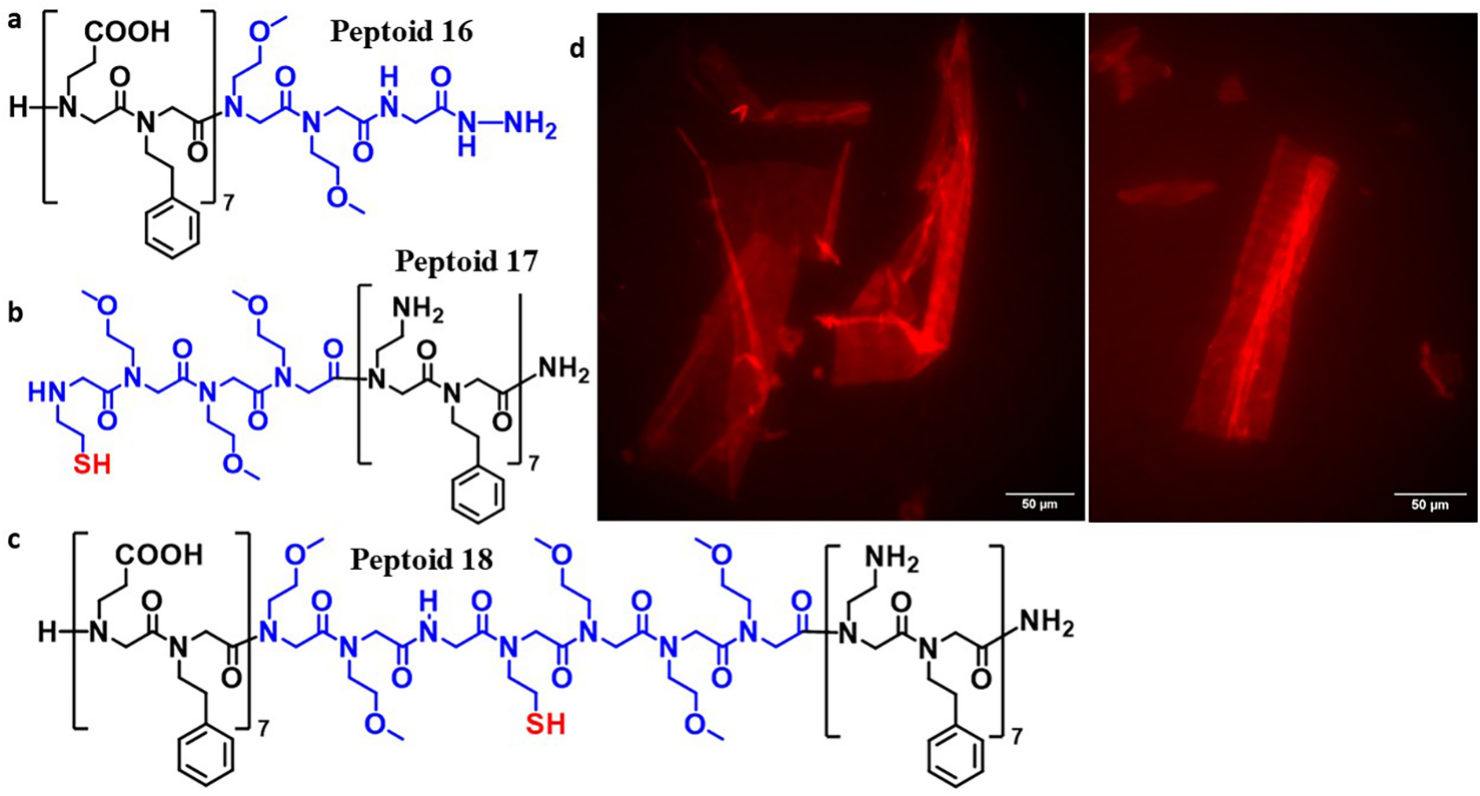
Formation of the expected product peptoid **18** from the ligation of peptoids **16** and **17**. (a) and (b) Sequences of peptoids **16** and **17**, respectively. (c) Sequence of ligation product peptoid **18**. Loop segment is shown in blue. (d) Fluorescence microscopy imaging of peptoid nanosheets formed from peptoid **18**. Nanosheets were stained with Nile Red dye on an inverted Zeiss Elyra 7 microscope.

We also designed a loop cassette system, in which the nanosheet-forming sequence is prepared via two ligations of three individual peptoid fragments: a positively charged N-terminal fragment, a central loop fragment, and a negatively charged C-terminal block (SI Figure 10). The C-terminal hydrazide is not reactive to the ligation conditions until it is oxidized with sodium nitrite and subsequently treated with the MPAA additive. A sequential ligation using these conditions can thus proceed in the N-to-C direction without a requirement for protecting groups at the C-termini. The intermediate product was isolated following the completion of the first ligation reaction and used as the C-terminal hydrazide peptoid in the second ligation reaction. We included an alkyne handle in the loop of this design to illustrate that additional functionalization of the sequence via CuAAC is possible. The desired ligation product was detected via analytical HPLC and LCMS following both ligation reactions, and the final product was rocked into nanosheets (SI Figures 11–13). Using this method, the efficient preparation of a library of loop-presenting peptoid nanosheets can be achieved via the sequential ligation of three peptoid fragments rather than a lengthy synthesis and challenging purification of the final sheet-forming oligomer. Shelf-stable flanking anchor blocks can be maintained in large quantities. This allows a variety of new loop-displaying sheet-forming peptoids to be formed and only requires the synthesis of short loop sequences.

As a representative analysis of the ligation of nanosheet-forming sequences, the peptoid nanosheet-forming product of the ligation between peptoids **14** and **15** was purified via preparative HPLC in 32% isolated yield. This yield falls somewhere between previously reported yields of other peptoid ligation protocols but is significantly higher than the reported serine/threonine ligation of a peptoid/protein hybrid, another ligation protocol resulting in an amide bond at the ligation site.^36^

One current limitation of this method is the necessity of using amino acid coupling chemistry for the C-terminal residue of the peptoid hydrazide. Only a sparse set of N-Fmoc, N-substituted glycine monomers are commercially available. This limits the chemical diversity readily available for that specific position. It is possible to synthesize desired Fmoc-protected peptoid monomers, however. This, in combination with the necessity for the thiol-containing monomer at the N-terminus of the other peptoid imposes some limitations on side chain diversity of adjacent residues at the ligation site. We also note that there is an off-target oxidation reaction when oxidizing a peptoid sequence with an amine-containing side chain at the N-terminal monomer to form an N-nitrosamine at the N-terminus (*vide supra*). These limitations are relatively minor however, especially when compared to other oligomer condensation techniques previously described.^8,19,34–36^

To our knowledge this is the first demonstration of chemical ligation between peptoid oligomers that generates native tertiary amide bonds at each ligation site. Peptoid chemists will now have access to a synthetic method that does not require a trade-off between sequence specificity and chain length. A synthetic method that only requires the purification of smaller, higher yielding fragments provides a convenient convergent workflow to access sequence-defined peptoid macromolecules. Most examples of long peptoid sequences are limited to only very high yielding side chain types, as the combination of diminishing yields from increased chain length and reduced yields from lower yielding side chain types makes obtaining sequence-specific peptoid macromolecules extremely challenging. We believe this method will allow for the preparation of peptoid macromolecular sequences that include low-yielding monomer types. As demonstrated in our synthesis of a loop-presenting peptoid nanosheet-forming sequence via sequential ligation of multiple fragments, this technique can be used to synthesize peptoid sequences modularly. We believe that a modular synthetic technique will greatly enhance the discovery rate of functional peptoid macromolecules along with novel peptoid secondary and tertiary structures. This modular synthetic approach will facilitate the combinatorial construction of diverse high-molecular weight peptoid sequences that can be screened for a desired function or the formation of stable hierarchical structures.

## Conclusions

We report the use of a variation of peptide native chemical ligation for the ligation and macrocyclization of peptoid fragments and the use of this protocol to synthesize 2D nanosheet-forming sequences. The use of C-terminal hydrazides as masked thioesters facilitates the preparation of peptoids to be used in NCL. These results demonstrate a simple and reliable method for the chemical conjugation of unprotected oligomer fragments. We expect that this strategy will be useful in the future preparation of sequence-defined peptoid macromolecules.

## Supplementary Material



## Notes

Analytical characterization of the reactions and compounds described above (PDF)
