# Supplementary material for: Native Chemical Ligation of Peptoid Oligomers
Source: Biochemistry. 2026 Mar 16;65(7):975–84. doi: 10.1021/acs.biochem.5c00833 (PMC13063420; doi:10.1021/acs.biochem.5c00833)
Supplement: Supplementary file 1 [file bi5c00833_si_001.pdf]

Supporting Information

Native Chemical Ligation of Peptoid Oligomers

Matthew R. Seraydarian[1,2], Michael D. Connolly[2], Ronald N. Zuckermann[2], and Kent Kirshenbaum[1]*

[1]Department of Chemistry, New York University, 100 Washington Square East, New York, New York 10003, United States

[2]The Molecular Foundry, Lawrence Berkeley National Laboratory, 1 Cyclotron Road, Berkeley, California 94720, United States

Corresponding Author email: kent@nyu.edu

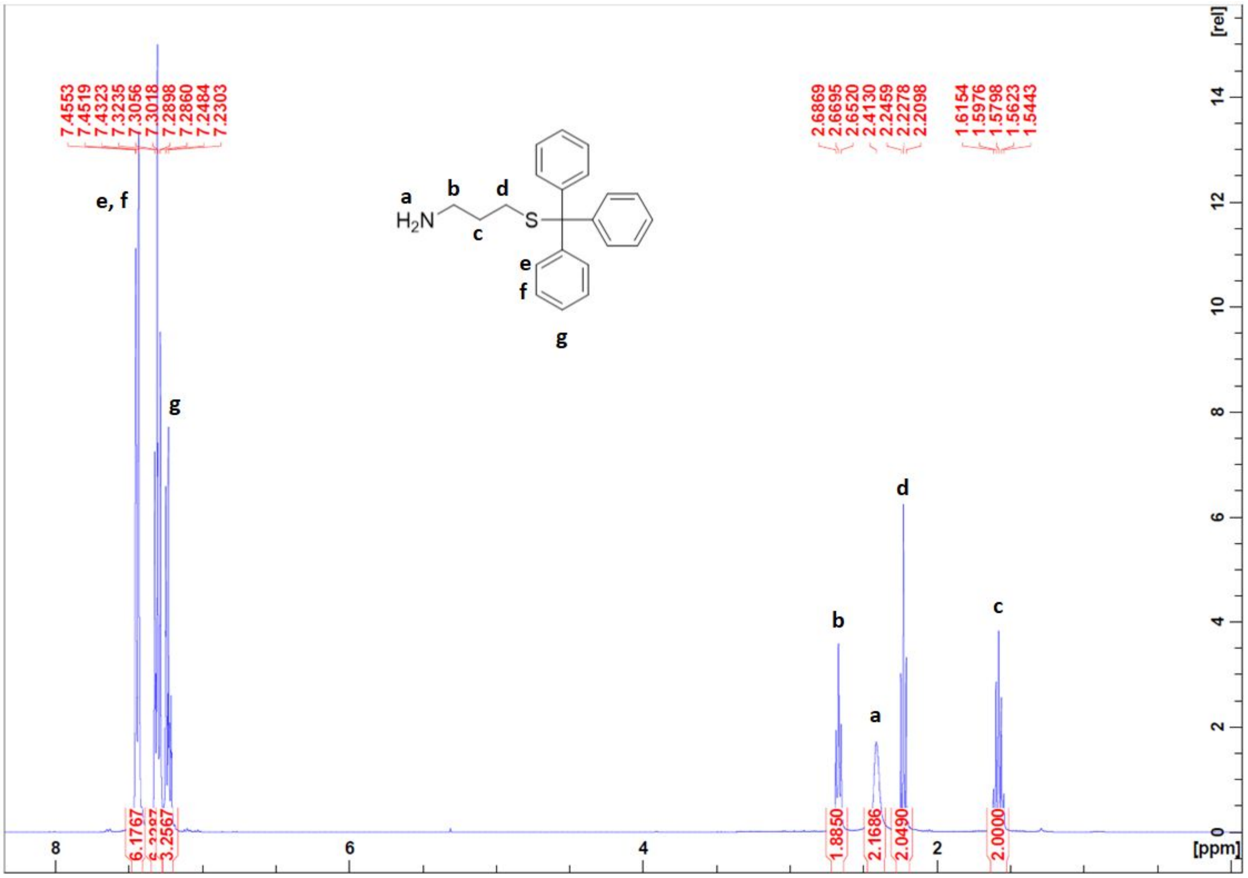

Supplemental Figure 1: 1H NMR of the synthesized 3-(tritylthio)-propylamine

Supplemental figures 2-4, shown below, detail the success of the portion of this project involving the ligation of short (<6 monomers) peptoid oligomers.

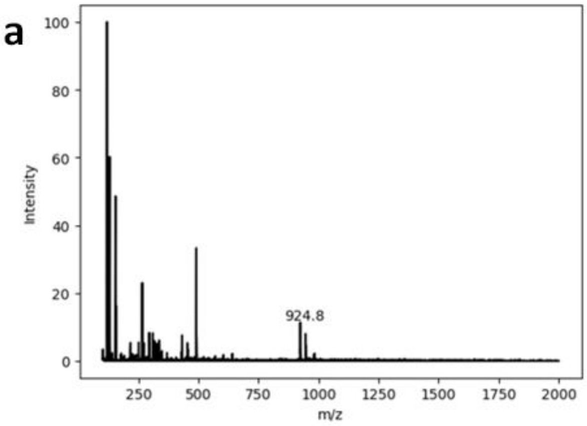

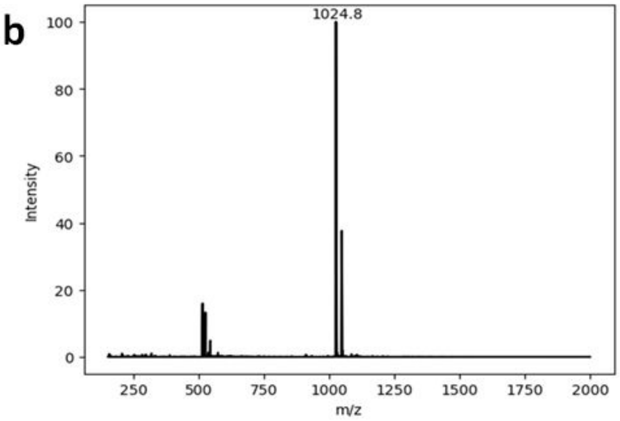

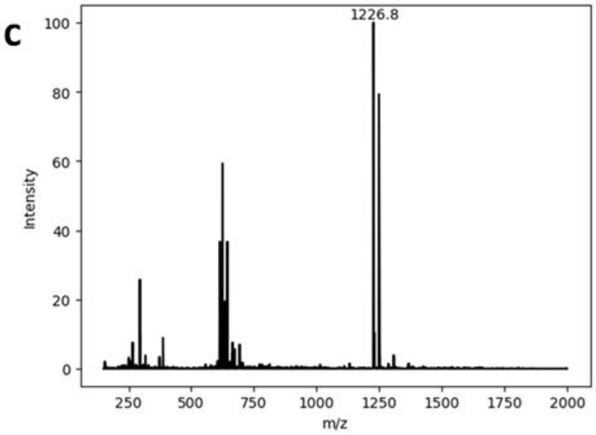

Supplemental Figure 2: Mass spectra of ligations of small model oligomers. (a) Mass spectrum of the ligated product H-(Npl-Npm)$_2$-Nte-(Npl)$_3$-NH$_2$ (peptoid **6**). (b) Mass spectrum of the ligated product H-Npm-(Nme)$_3$-Sar- Nsp-Npl-Npm-Nme-NH$_2$ (peptoid **9**). (c) Mass spectrum of the ligated product H-Npm-(Nme)$_3$-Sar- Nte-Npl-Nte-Npm-Nme-Npl-NH$_2$ (peptoid **11**). Spectra (b) and (c) were obtained via a column separation method. [M+Na] values are visible in all spectra. The ligation reaction is performed in a aqueous buffer containing sodium, so [M+Na] values are frequently seen.

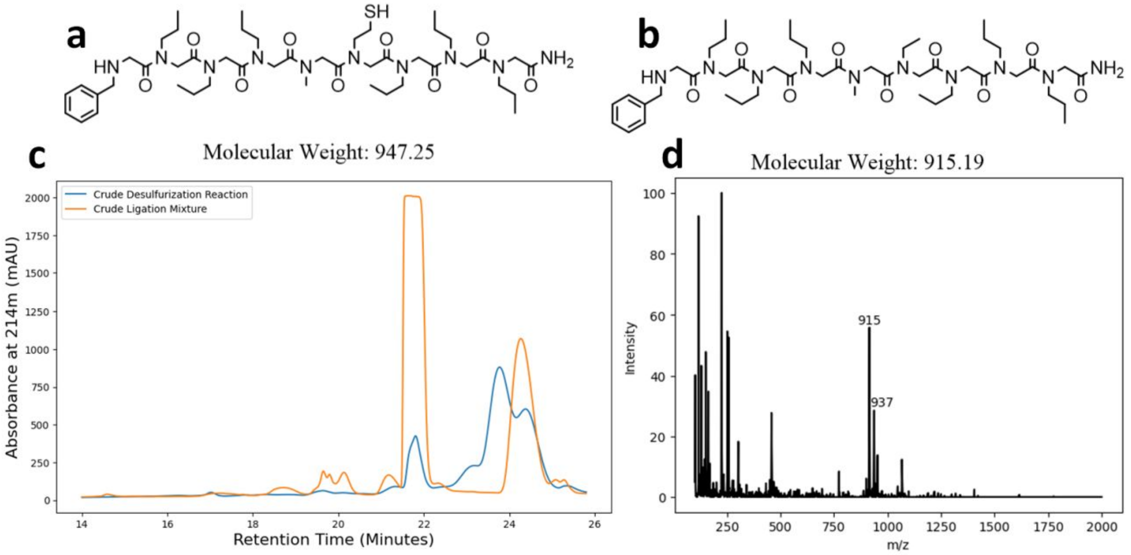

Supplemental Figure 3: (a) and (b): Structures of the initial ligation product (peptoid **3**) and the ligation product after desulfurization (peptoid **3DS**), respectively. (c) Analytical HPLC traces of the crude ligation reaction and crude desulfurization reaction overlayed. In this graph, the MPAA additive is found just before 22 minutes (this method had a 5-minute delay compared to the one used in supplemental figure 2). The ligation product is seen just after 24 minutes, and the desulfurization product is seen just before 24 minutes. From this analysis it is clear that desulfurization did not complete in the time the reaction ran (4 hours), as both ligation product and desulfurization product are seen. (d) MS of the desulfurization product.

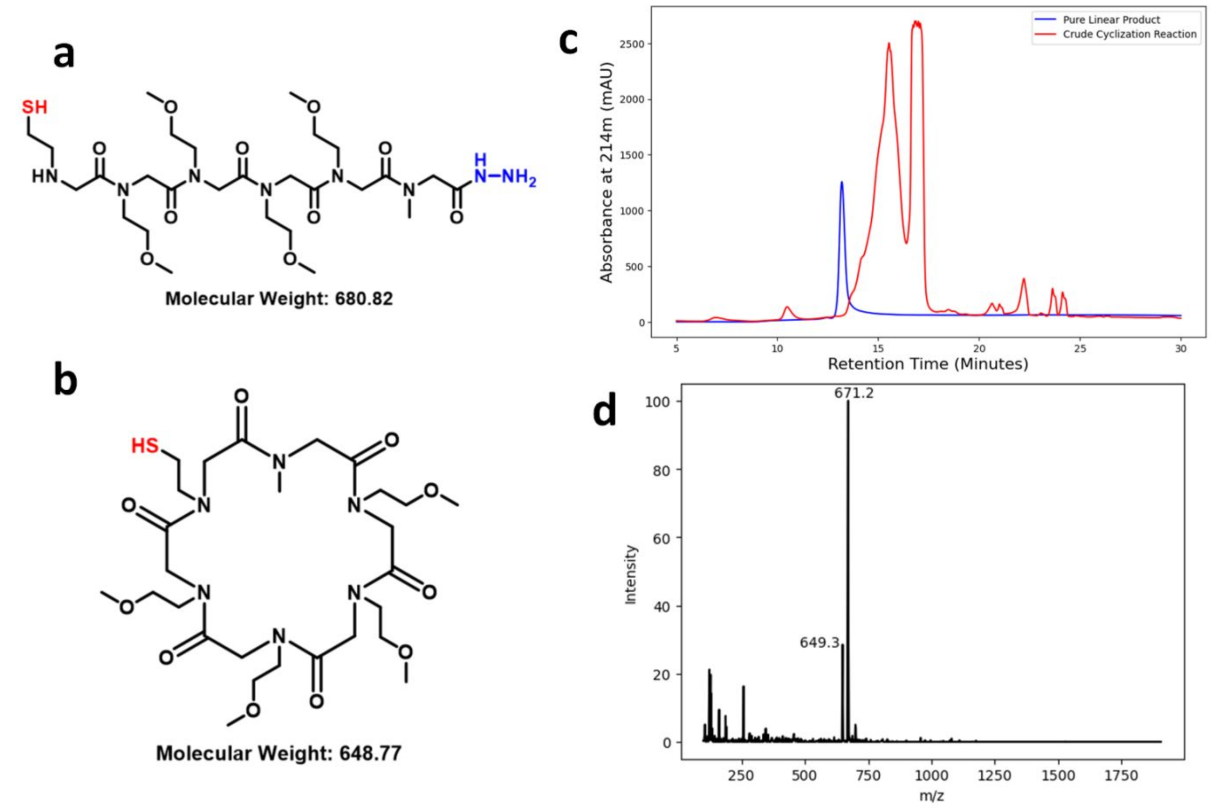

Supplemental Figure 4: (a) and (b): Structures of the linear precursor and cyclic product, respectively. (c) Analytical HPLC traces of the pure linear precursor and the crude cyclic reaction overlayed. In the crude cyclic reaction, the product is found shortly after 15 minutes. The large peak at 17 minutes shortly after the product is the MPAA additive, which is used in high excess. (d) MS of the cyclized product. The most abundant mass species is the [M+Na] 671.2 mass. It is common to see the [M+Na] as the most abundant species in the MS of peptoid macrocycles.

Amines used to synthesize their corresponding N-substituted glycine monomers are shown below in SI table 1.

Supplemental Table 1: N-substituted glycine monomers used during this study, and the corresponding amines used to synthesize them via solid phase sub-monomer synthesis

**Monomer Name**	**Sub-monomer Amine**
N-(2-methoxyethyl)glycine	2-methoxyethylamine
N-(propyl)glycine	propylamine
N-(phenylmethyl)glycine	benzylamine
N-(2-mercaptoethyl)glycine	2-(tritylthio)ethanamine
N-(3-mercaptopropylglycine)	3-(tritylthio)propylamine
N-(2-phenylethyl)glycine	2-phenylethylamine
N-(2-aminoethyl)glycine	N-Boc-ethylenediamine
N-(2-carboxyethyl)glycine	β-Alanine *tert*-Butyl Ester Hydrochloride
N-(propargyl)glycine	propargylamine

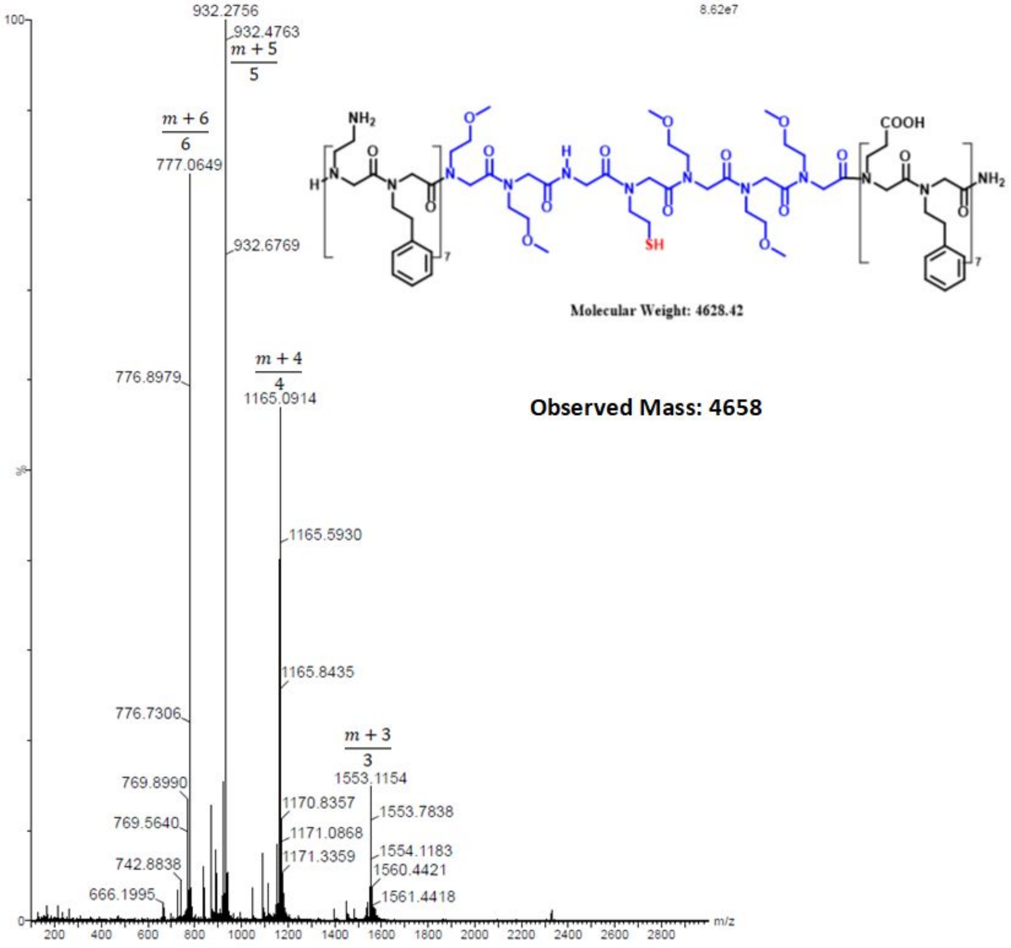

Supplemental Figure 5: Mass spectrum showing a mass ~29 Da larger than the mass of the expected ligation product of peptoids **14** and **15**

Further Investigation of +29 mass on oxidation of peptoids with a primary amine-containing side chain at the N-terminus:

We sought to investigate what causes the +29 mass addition when oxidizing the positively charged portion of the nanosheet-forming sequence. We hypothesized that the issue is associated with the N-terminus and the positively charged primary amine as the N-terminal peptoid monomer. To test this, we designed two peptoid trimers, one with the primary amine side chain at the N-terminus, and one with this side chain at the C-terminus, shown below in SI figure 6. Each of these peptoids were synthesized and cleaved from resin but not purified further. Upon dissolution in ligation buffer and oxidation of each of these peptoids with $NaNO_2$, only the peptoid with the amine-containing side chain at the N-terminus showed significant conversion to the +29 mass.

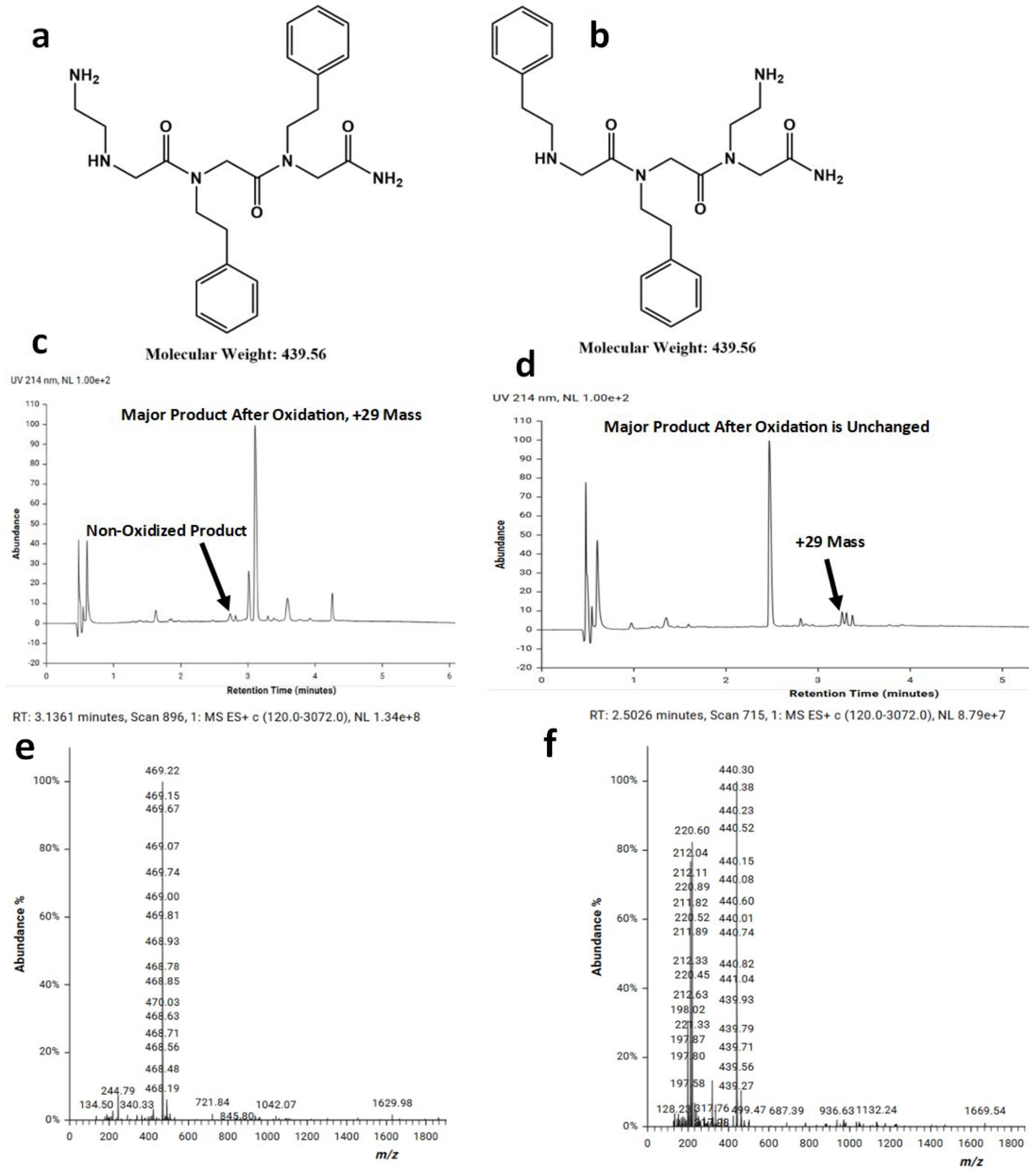

Supplemental Figure 6: (a) and (b) Structures of peptoid trimers synthesized to test the effect of the position of the Nae side chain on whether the +29 Da mass is present. (c) and (d) UPLC

traces of structures (a) and (b) after oxidation with $NaNO_2$, respectively. (e) and (f) MS of the major product peaks from the UPLC traces (c) and (d), respectively.

We compared the FTIR spectra of the pure peptoid shown in SI figure 6(a), and its oxidized variant that results in the +29 mass, shown below in SI figure 7. The most notable differences between the spectra are the new peaks at ~1450cm^{-1} and ~950cm^{-1}. N-nitrosamines have been previously characterized in dipeptides containing N-terminal prolines by IR bands at 1430cm^{-1}, and in small organic compounds by bands at 1425-1500cm^{-1} and 925-1150cm^{-1}, substantiating the notion that the product forming from the reaction of our peptoids with $NaNO_2$ is a nitrosamine[1,2]. UV-Vis spectroscopy was also consistent with the presence of an N-nitrosamine product, as indicated by the presence of absorbance bands at 240nm and 350nm[1].

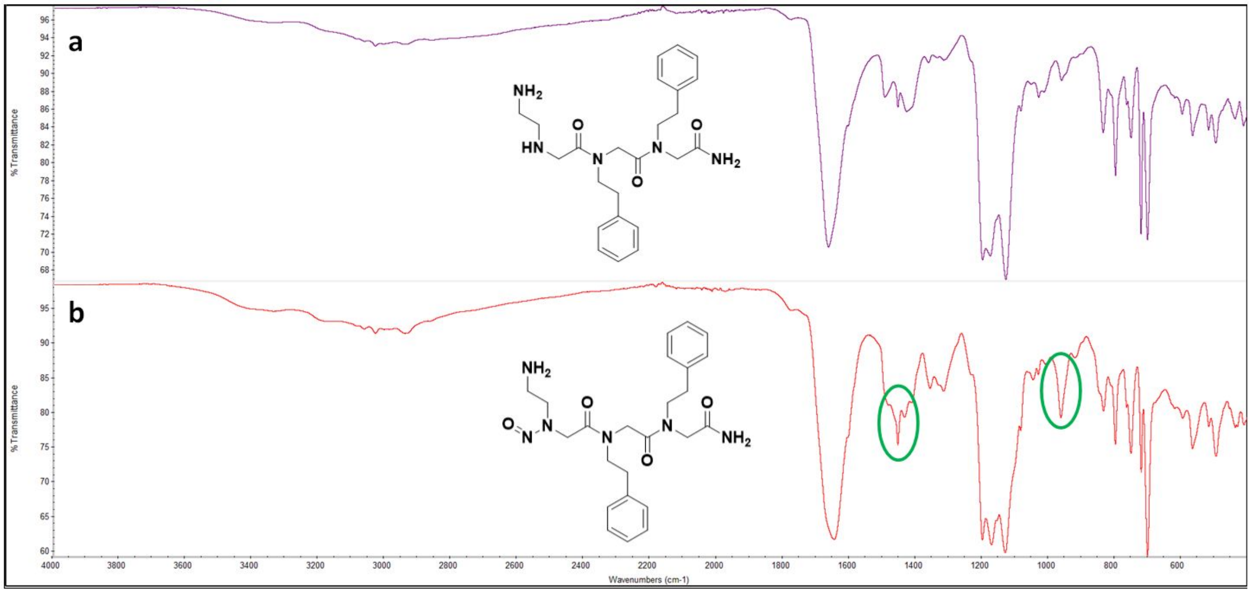

Supplemental Figure 7: FTIR spectra of the pure peptoid trimer and the proposed N-nitrosamine variant. (a) FTIR spectrum and chemical structure of the peptoid trimer original shown in SI

figure 6(a). (b) FTIR spectrum and chemical structure of the variant of the peptoid trimer containing an N-nitrosamine at its N-terminus. New IR bands are circled in green.

Following up on this, we synthesized the peptoid trimers shown below in SI figure 8. The sequence with the free N-terminus (SI figure 8 (a)) showed only minimal oxidation to the N-nitrosamine species as a minor product as indicated by UPLC-MS. The trimer with the formylated N-terminus (SI figure 8 (b)) showed no conversion to the N-nitrosamine species, even as a minor product. The dependence of side chain length and necessity of the N-terminus being free on the formation of the N-terminal N-nitrosamine suggests a cyclic structure involving the side chain primary amine is forming and is a source of stability for the structure.

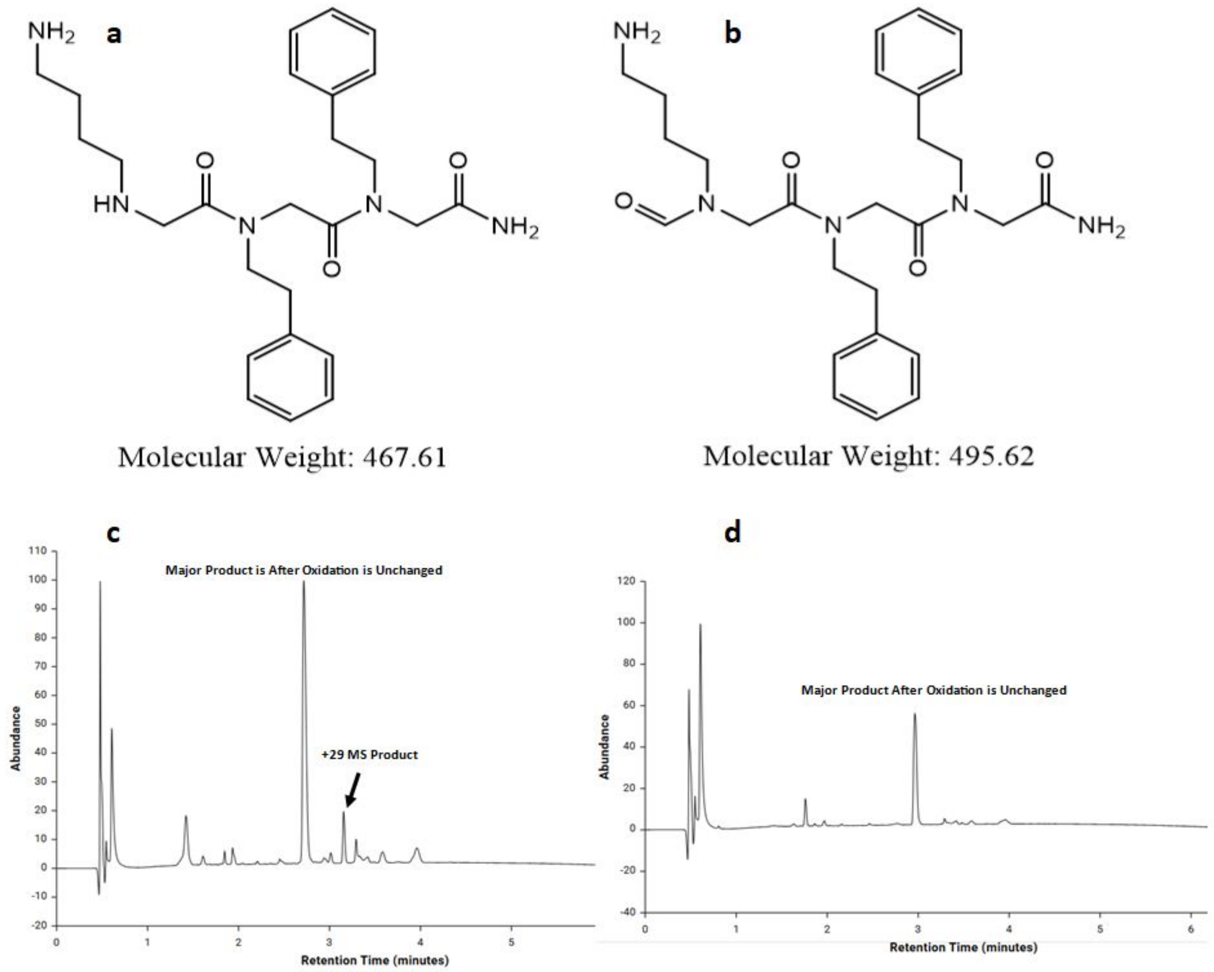

Supplemental Figure 8: (a) and (b) Structures of peptoid trimers with longer primary amine-containing side chains synthesized. (c) and (d) UPLC traces of structures (a) and (b) following oxidation with $NaNO_2$

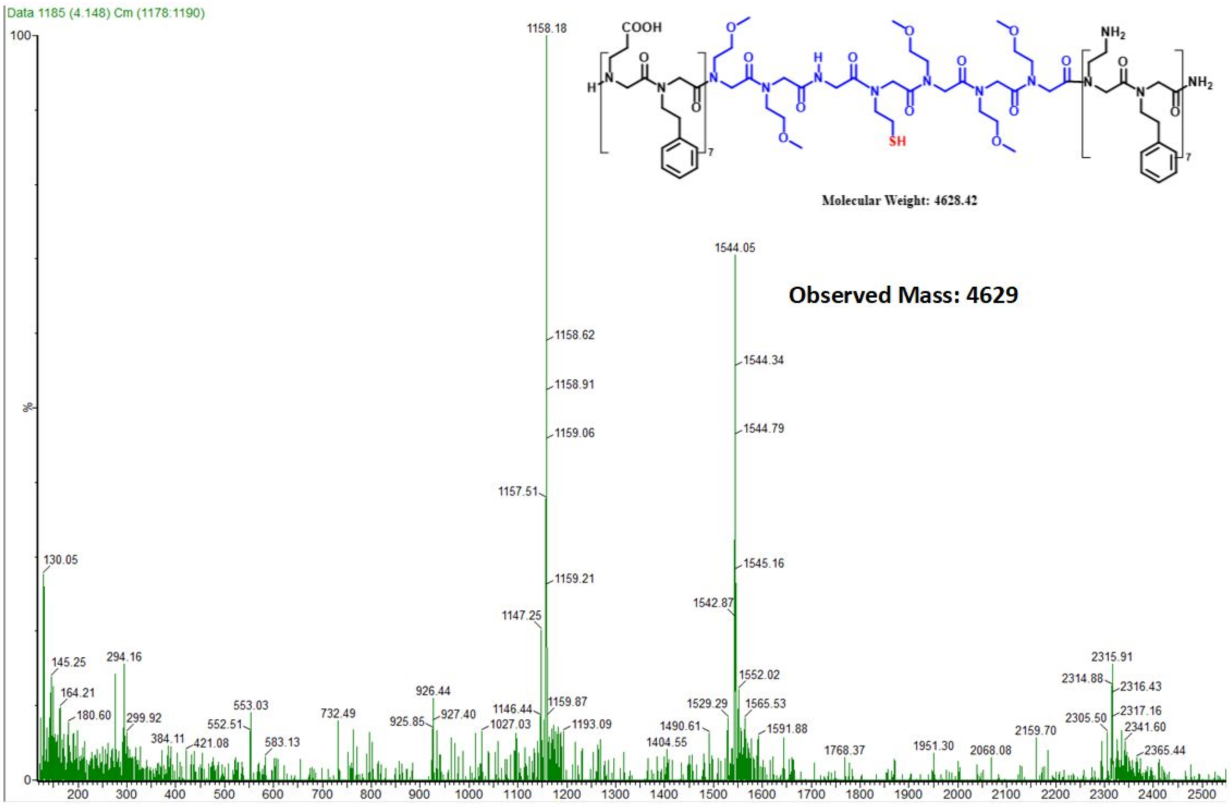

Supplemental Figure 9: Mass spectrum of the expected ligation product peptoid **18** with no additional mass.

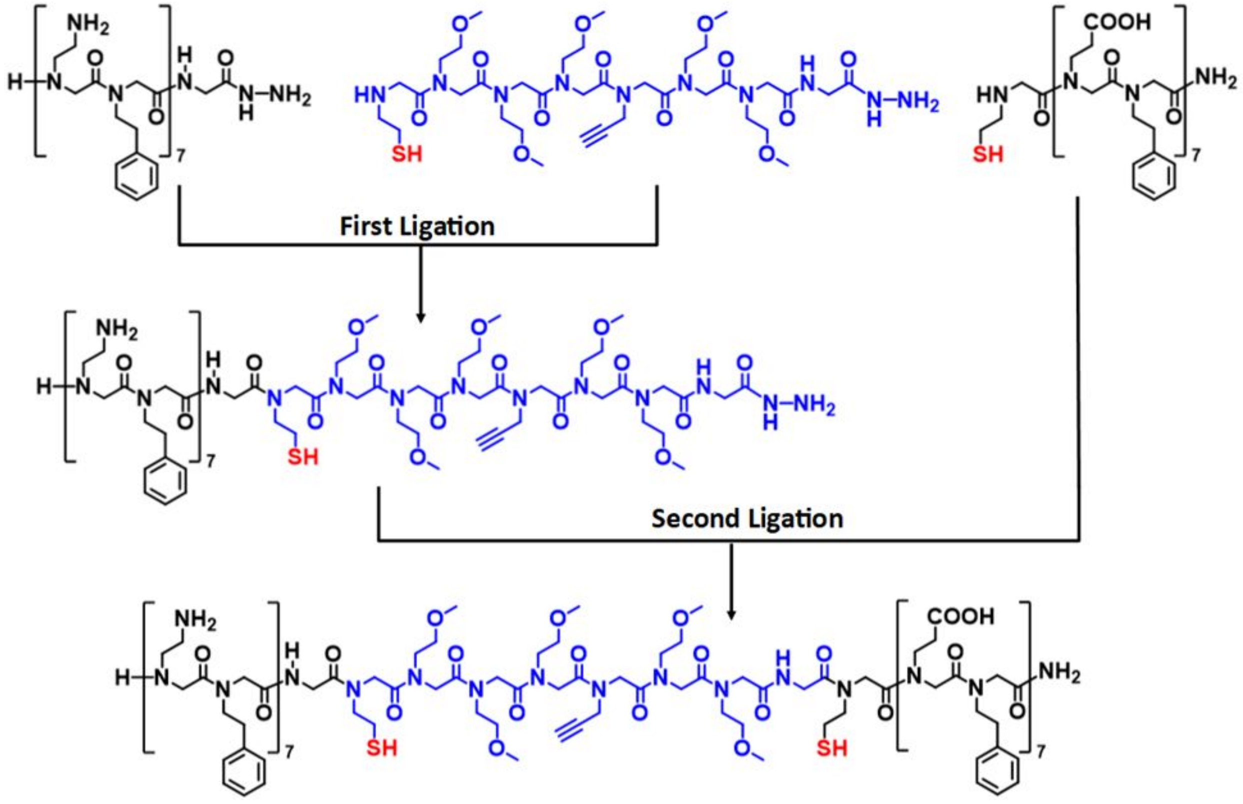

Supplemental Figure 10: Scheme depicting the attempted sequential ligation of a nanosheet-forming sequence containing an alkyne in the loop.

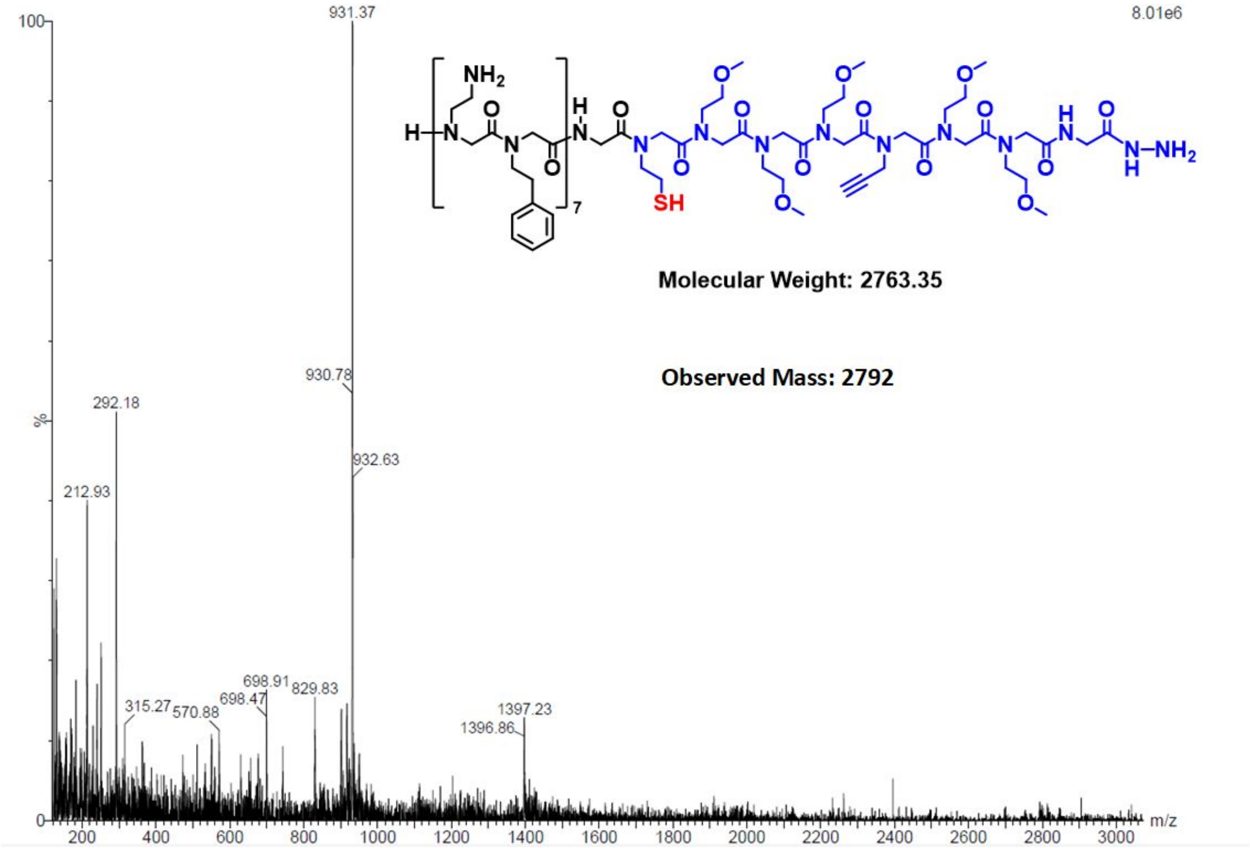

Supplemental Figure 11: Mass spectrum displaying a mass ~29 Da larger than the expected intermediate ligation product.

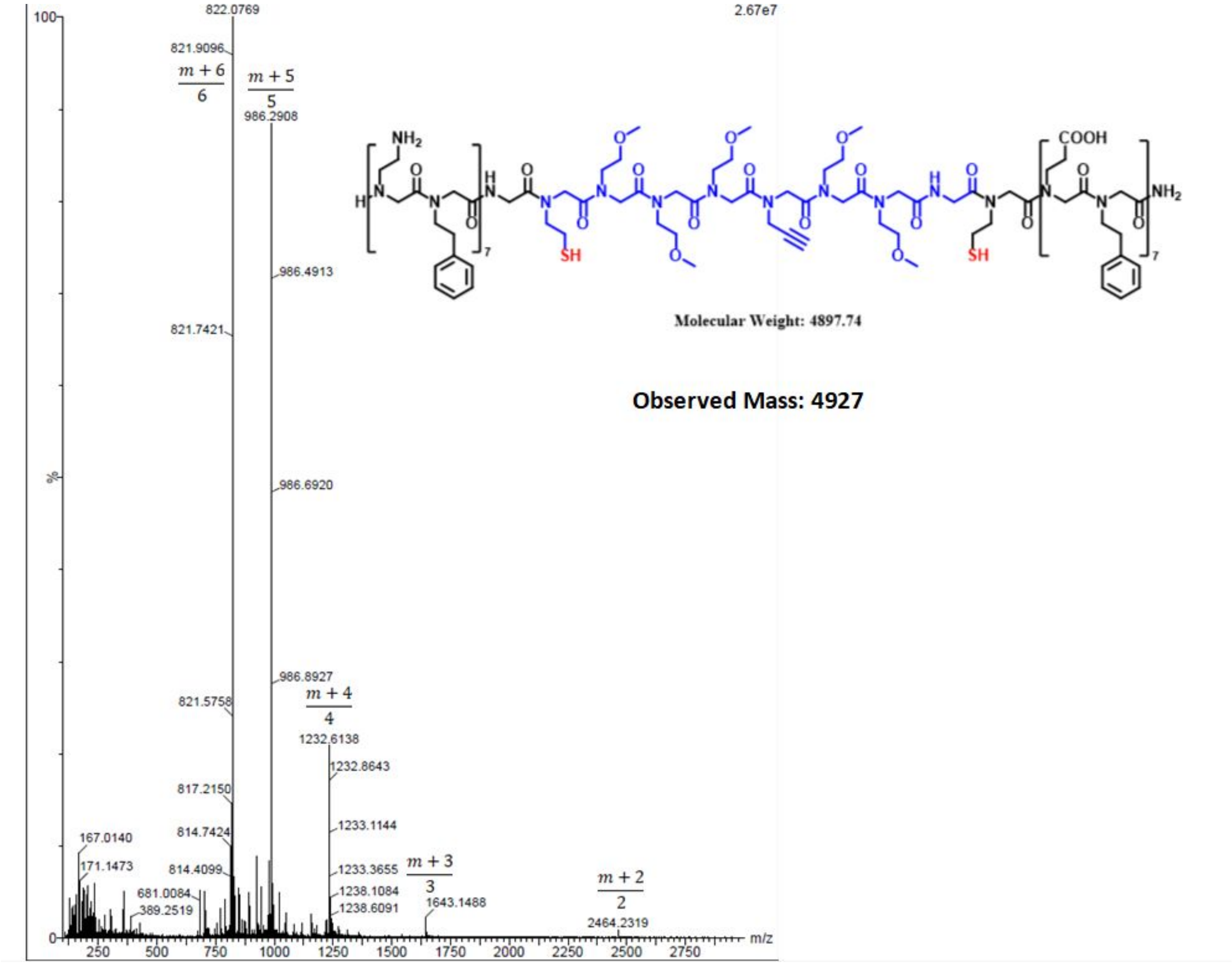

Supplemental Figure 12: Mass spectrum displaying a mass ~29 Da larger than the expected final sequential ligation product.

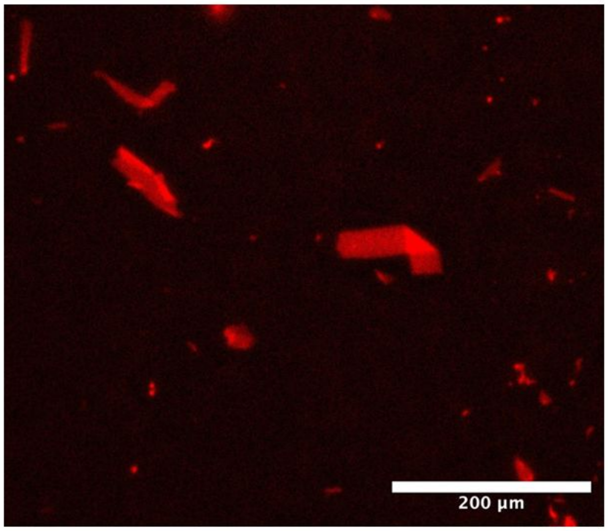

Supplemental Figure 13: Nanosheets formed from the two-step sequential ligation product.

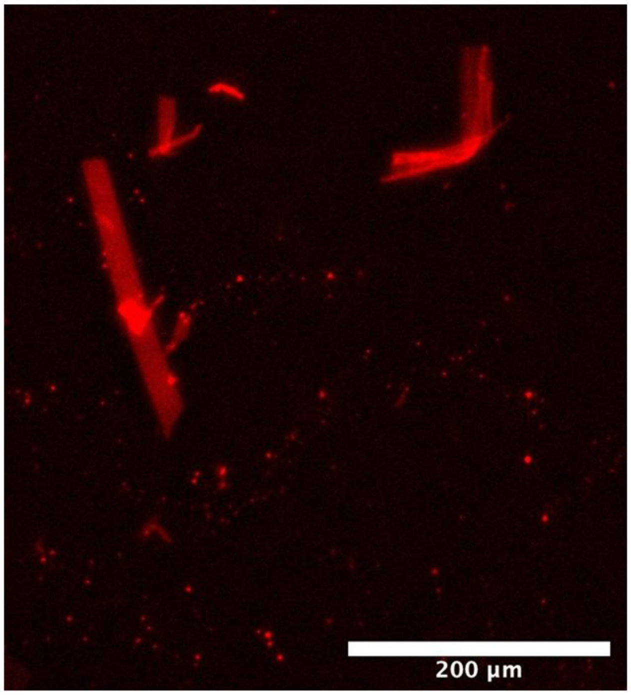

References:

1. Kubacka, W.; Libbey, L. M.; Scanlan, R. A. Formation and chemical characterization of some nitroso dipeptides. N-Terminal in proline. *Journal of Agricultural and Food Chemistry* **1984**, *32* (2), 401-404.
2. Williams, R.; Pace, R.; Jeacocke, G. Applications of solvent effects—I: The spectra of secondary nitrosamines. *Spectrochimica Acta* **1964**, *20* (2), 225-236.
